# Whole-genome sequencing and molecular analysis of carbapenem-resistant *Escherichia coli* from clinical samples collected in the framework of the EURGen-Net CCRE survey, Italy 2019

**DOI:** 10.3389/fmicb.2026.1875003

**Published:** 2026-07-24

**Authors:** Maria Giufrè, Giulia Errico, Maria Del Grosso, Sara Giancristofaro, Michela Pagnotta, Fabio D’Ambrosio, Simone Iacchini, Sophia David, Alma Brolund, Anke Kohlenberg, Annalisa Pantosti, Monica Monaco, the Italian CCRE working group

**Affiliations:** 1 Department of Infectious Diseases, Istituto Superiore di Sanità, Rome, Italy; 2 Centre for Genomic Pathogen Surveillance, Pandemic Sciences Institute, University of Oxford, Oxford, United Kingdom; 3 Department of Communicable Disease Control and Preparedness, Public Health Agency of Sweden, Solna, Sweden; 4 European Centre for Disease Prevention and Control, Stockholm, Sweden

**Keywords:** carbapenem resistance, *Escherichia coli*, genomic analysis, molecular epidemiology, WGS

## Abstract

**Introduction:**

Carbapenem-resistant (CR)-Enterobacterales are a serious threat to public health worldwide. However, there are limited surveillance data on the molecular epidemiology of CR-*Escherichia coli*. To address this need, the European Centre for Disease Prevention and Control (ECDC) investigated the molecular epidemiology of CR-*E. coli* in the carbapenem- and/or colistin-resistant Enterobacterales (CCRE) survey launched in 2019, involving 37 European countries, including Italy.

**Methods:**

In 2019, 46 acute-care hospitals in Italy were enrolled to collect the first 10 non-duplicated consecutive isolates of carbapenem non-susceptible *Klebsiella pneumoniae* specie complex or *E. coli* isolates and 10 carbapenem-susceptible comparator isolates of the same species. Each isolate was subjected to antibiotic susceptibility testing. WGS analysis was performed using Illumina technology to determine the ST and to detect resistance genes, virulence factors, and plasmid content.

**Results:**

A total of 61 *E. coli* isolates were collected from 17 hospitals: 17 CR-*E. coli* isolates (from urine, 41.2%, and blood, 23.5%) and 44 carbapenem-susceptible (CS)-isolates (from blood, 43.2%, and urine, 38.6%). All the CR-*E. coli* isolates were resistant to amoxicillin/clavulanic acid, cefepime, ceftazidime and piperacillin/tazobactam; three were resistant to ceftazidime/avibactam and all were susceptible to amikacin and tigecycline. MLST analysis showed the presence of unrelated lineages: CR-*E. coli* isolates belonged to 8 different STs, with ST131 predominant (58.8%). All the CR-*E. coli* isolates carried one carbapenemase: KPC-3 (10 isolates), KPC-2 (three), NDM-5 (two), VIM-1 and OXA-181 (one each). Among CS-*E. coli* isolates, the highest resistance rates were observed for amoxicillin/clavulanic acid (36.4%) followed by ciprofloxacin, and trimethoprim/sulfamethoxazole, and ST131, carrying CTX-M-15, was the predominant clone.

**Discussion:**

CR-*E. coli* clones from infection showed a heterogeneous genetic background, ST131 being the most frequent. KPC was the most common carbapenemase but the presence of *E. coli* isolates carrying NDM-5 is worrisome, as it results in resistance to several novel beta-lactam-beta-lactamase combinations. Our study underlines the importance of genomic surveillance to monitor and prevent the spread of high-risk clones causing difficult-to-treat infections.

## 1 Introduction

Carbapenem-resistant Enterobacterales have become a serious threat to public health worldwide, especially for *Klebsiella pneumoniae* and to a lesser extent for *Escherichia coli* (Cummins et al., 2021). *E. coli* is a Gram-negative bacterium found as commensal in the human gut (Pitout, 2012), nonetheless, it is one of the main causes of infectious diseases in both hospital and community settings (Kaper et al., 2004) and the management of such infections is often hampered by antibiotic resistance (Gajdács et al., 2021). Third-generation cephalosporin-resistant and carbapenem-resistant (CR) *E. coli* are now regarded as a major public health issue, included in the WHO critical-priority list of resistant bacteria pathogens for the development of new antibiotics (World Health Organization, 2024; Tacconelli et al., 2018; Huang et al., 2024). CR-*E. coli* can lead to severe infections, including intra-abdominal infections, pneumonia, urinary tract infections, and device-associated infections.

In Italy, the percentage of CR-*E. coli* has remained stable in the last years, in line with the European trend (0.3% in 2024) (European Centre for Disease Prevention and Control, 2025). The principal resistance mechanism to carbapenems in CR- *E. coli* isolates is the production of carbapenemase enzymes (Grundmann et al., 2017). Resistance genes are frequently located on mobile genetic elements such as plasmids and transposons, which can lead to a more rapid dissemination of resistance through horizontal gene transfer (Kopotsa et al., 2019). In recent years, novel antibiotics have been introduced in clinical practice to treat CR-*E. coli* infections, including the new *β*-lactam/*β*-lactamase inhibitor combinations such as ceftazidime-avibactam, meropenem-vaborbactam, and imipenem-relebactam (Yahav et al., 2020; Kiratisin et al., 2021). However, emerging resistance to these agents has already been reported, primarily due to mutations in *β*-lactamase genes, porin loss, or efflux pump overexpression, which may compromise their long-term effectiveness (Yahav et al., 2020; Kiratisin et al., 2021).

Since there are limited data on the molecular epidemiology of CR-*E. coli*, in 2019 the European Centre for Disease Prevention and Control (ECDC) launched the carbapenem- and/or colistin-resistant Enterobacterales (CCRE) survey, built on the previous European Survey of carbapenemase-producing Enterobacteriaceae (EuSCAPE) conducted in 2013–2014 (Grundmann et al., 2017). The CCRE survey involving 37 European countries, including Italy, was initiated with the aim of implementing EU-level genomic-based surveillance of high-risk *K. pneumoniae* and *E. coli* clones (European Centre for Disease Prevention and Control (ECDC), 2018).

In this framework, we only focused the analysis on CR-*E. coli* isolates collected from hospital microbiology laboratories participating in the national Surveillance of Antibiotic-Resistance Network AR-ISS, with the aim to characterise them at genomic level by investigating genetic diversity and antibiotic resistance profiles.

## 2 Materials and methods

During 2019, between May and October, in the framework of the CCRE survey conducted by ECDC, 46 acute-care AR-ISS hospitals were enrolled in Italy (two hospital per Nomenclature of Territorial Units for Statistics, NUTS, level-2 regions) distributed throughout the country (European Centre for Disease Prevention and Control (ECDC), 2018). During the study period, each hospital microbiology laboratory was asked to collect the first 10 non-duplicated consecutive isolates of carbapenem-resistant or -susceptible increased exposure (CR) *K. pneumoniae* species complex or *E. coli*, exhibiting a meropenem and/or imipenem MIC >2 mg/L, and/or ertapenem MIC >0.5 mg/L. Isolates obtained through routine laboratory diagnostics from clinical specimens (e.g., blood, urine, sputum, and wound secretions) as well as colonization samples from consecutive patients were included in the study. The first following 10 carbapenem-susceptible (CS) isolates of the same species were also collected and included in the analysis as comparator isolates, to enable characterisation of the baseline genomic population structure. Hospital microbiology laboratories were also asked to collect patient epidemiological and clinical data (age, sex, hospital ward, previous hospital admission or residence in a long-term care facility in the last 6 months, hospital acquisition or community onset colonisation/infection defined based on sample collection from patients with an acute care hospital stay of more or less than 48 h post-admission).

Bacterial identification and *in vitro* susceptibility testing were performed by the participating laboratories using routine laboratory methods. Collected isolates were sent for confirmation analysis to the National Reference Laboratory (NRL) for Antibiotic Resistance at Istituto Superiore di Sanità (ISS, Rome, Italy), designated as the National Expert Laboratory (NEL) for the study purpose. At the NRL, isolates were subjected to antibiotic susceptibility testing (AST) using microtitration plates (Merlin Diagnostika, Berlin, Germany) and agar dilution for fosfomycin by AD fosfomycin panel (Liofilchem, Roseto degli Abruzzi, Italy). AST results were interpreted in accordance with 2019 EUCAST clinical breakpoints (The European Committee on Antimicrobial Susceptibility Testing, 2019). *E. coli* ATCC 25922 was used as the quality control strain.

Whole genome sequencing of the characterised isolates was performed at the Wellcome Sanger Institute using the Illumina NovaSeq6000 platform with 150 bp reads. Sequencing reads were assembled into contigs using SPAdes v3.10.0 (Bankevich et al., 2012) with the “--careful” flag provided and the “--cov-cutoff” flag set to “auto.” In silico analysis of the assembled contigs was performed at ISS using the Achtman MLST scheme, CHTyper based on *fumC/fimH/* genes, PlasmidFinder and ResFinder at the Center for Genomic Epidemiology (CGE) server^1^, and AMRFinderPlus at the National Center for Biotechnology Information (NCBI) server^2^ to determine the sequence type (ST) and to detect plasmid and resistance genes content. Moreover, Pathogenwatch was used to obtain Phylogroup, Serotype and cgMLST of all the *E. coli* genome sequences.^3^ Identification of virulence genes related to *E. coli* was performed by ABRicate using Virulence Factor database at Galaxy EU.^4^ A core genome single-nucleotide polymorphism (SNP)-based phylogeny was generated by CSI Phylogeny 1.4 at the CGE,^5^ using the complete genome of *E. coli* strain K12 (accession no. NC_000913.2) as a reference strain. The resulting maximum-likelihood phylogenetic tree in Newick format was visualized using Microreact^6^ (Argimón et al., 2016). A 17 SNP cut-off was applied to all pairs of *E. coli* genome sequences to identify putative nosocomial transmission events (Ludden et al., 2021).

## 3 Results

### 3.1 Patients’ characteristics

Seventeen out of 46 enrolled hospitals provided a total of 61 *E. coli* isolates including 17 CR- and 44 CS-*E. coli* isolates. CR-*E. coli* isolates were mainly collected from the older age groups with 71.3 and 75 years as the mean and median ages, respectively (range 52–90 years, Table 1); 9 were female (52.9%) and all were inpatients except one (94.1%). The CS-*E. coli* isolates were obtained from patients with 62.5 and 71 years as the mean and median ages, respectively (range 1–97 years, Table 1); 21 were female (47.7%) and 39 were inpatients (88.6%). CR-*E. coli* isolates were obtained mainly from cases classified as infections (predominantly urinary tract and bloodstream infections, 41.2 and 23.5%, respectively, Table 1) and were almost all from medical units (94.1%, Table 1). CS-*E. coli* isolates were obtained mainly from bloodstream and urinary tract infections (43.2 and 38.6%, respectively, Table 1), and from medical units in 70.5% of cases (Table 1). Hospital acquisition was significantly associated with CR-*E. coli* isolates (76.5% in CR-*E. coli* isolates vs. 43.2% in comparator isolates, *p* = 0.0196), and previous hospitalization within 6 months was a risk factor associated with CR-*E. coli* isolates (52.9% in CR-*E. coli* isolates vs. 20.5% in comparator isolates, p = 0.0071).

**Table 1:** Epidemiological characteristics for *E. coli* isolates.

*N* (%)	Overall	Carbapenem-resistant *E. coli*	Carbapenem-susceptible *E. coli*
	*N* = 61	*N* = 17	*N* = 44
Gender			
Male	31 (50.8)	8 (47.1)	23 (52.3)
Female	30 (49.2)	9 (52.9)	21 (47.7)
Age (%)			
0–19	3 (4.9)	0	3 (6.8)
20–39	6 (9.8)	0	6 (13.6)
40–59	13 (21.3)	4 (23.5)	9 (20.5)
60–69	6 (9.8)	3 (17.6)	3 (6.8)
70–79	17 (27.9)	6 (35.3)	11 (25.0)
80–89	10 (16.4)	3 (17.6)	7 (15.9)
≥90	6 (9.8)	1 (5.9)	5 (11.4)
Hospitalisation status (%)			
Outpatient	6 (9.8)	1 (5.9)	5 (11.4)
Inpatient	55 (90.2)	16 (94.1)	39 (88.6)
Hospital ward (%)			
Medical	47 (77.0)	16 (94.1)	31 (70.5)
Intensive care	4 (6.6)	0	4 (9.1)
Surgical	5 (8.2)	1 (5.9)	4 (9.1)
Other	5 (8.2)	0	5 (11.4)
Site of infection (%)			
Bloodstream	23 (37.7)	4 (23.5)	19 (43.2)
Urinary tract	24 (39.3)	7 (41.2)	17 (38.6)
Lower respiratory tract	4 (6.6)	1 (5.9)	3 (6.8)
Intra-abdominal	4 (6.6)	1 (5.9)	3 (6.8)
Skin and soft tissue	5 (8.2)	3 (17.6)	2 (4.5)
Other	1 (1.6)	1 (5.9)	0
Hospital/community acquisition (%)			
Hospital-acquired	32 (52.5)	13 (76.5)	19 (43.2)
Community-onset	23 (37.7)	2 (11.8)	21 (47.7)
Missing	6 (9.8)	2 (11.8)	4 (9.1)
Previous hospitalisation within 6 months (%)			
Same hospital	18 (29.5)	9 (52.9)	9 (20.5)
Another hospital	2 (3.3)	1 (5.9)	1 (2.3)
No hospitalisation	6 (9.8)	0	6 (13.6)
Missing	35 (57.4)	7 (41.2)	28 (63.6)
Previous residence in a long-term care facility (%)			
No	14 (23.0)	8 (47.1)	6 (13.6)
Yes	3 (4.9)	2 (11.8)	1 (2.3)
Missing	44 (72.1)	7 (41.2)	37 (84.1)

### 3.2 AST results

**Figure 1:**
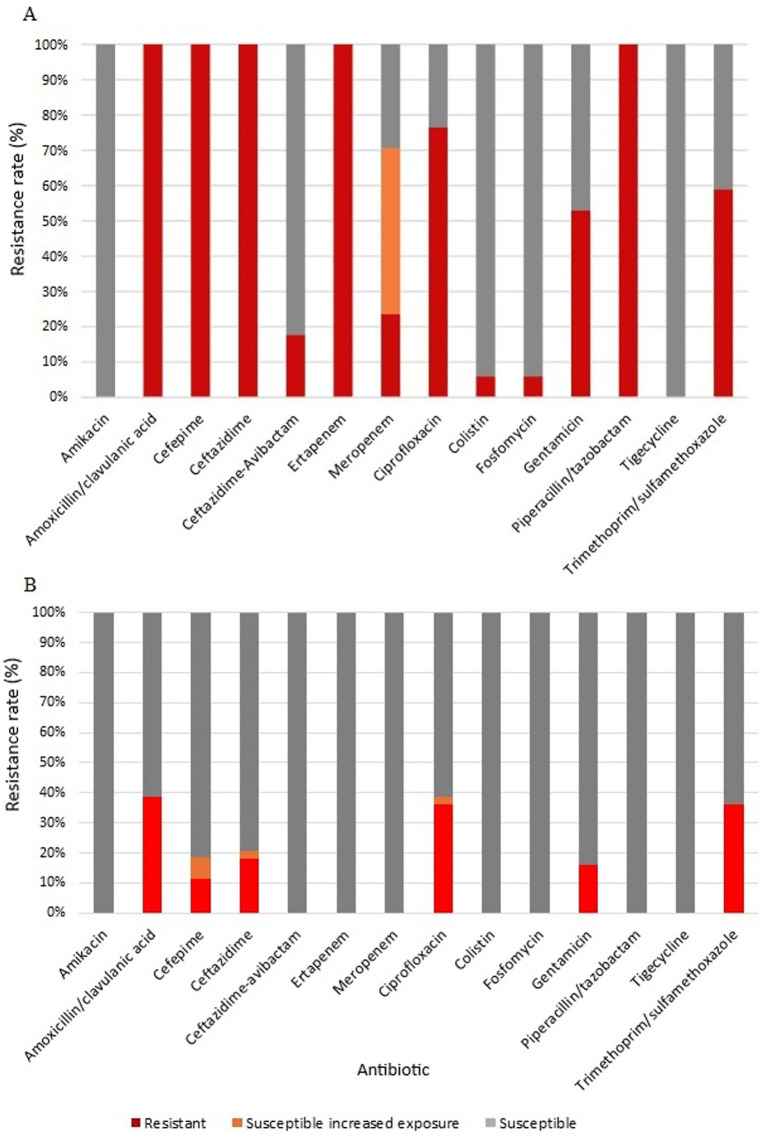
Antimicrobial susceptibility profiles of 17 CR-*E. coli* isolates **(A)** and of 44 CS-*E. coli* isolates **(B)**.

Antibiotic susceptibility testing of CR-*E. coli* isolates showed that all isolates were resistant to at least one (ertapenem) of the tested carbapenems (Figure 1A) in line with the inclusion criteria, while all the 44 CS-*E. coli* isolates were confirmed susceptible to carbapenems (Figure 1B). All the CR-*E. coli* isolates were multidrug-resistant (MDR) and exhibited resistance to amoxicillin/clavulanic acid, cefepime, ceftazidime and piperacillin/tazobactam (Figure 1A). Most CR-*E. coli* isolates were also resistant to ciprofloxacin (13/17, 76.5%) and trimethoprim/sulfamethoxazole (10/17, 58.8%), while over half of the isolates were also resistant to gentamicin (9/17, 52.9%). The rate of colistin and fosfomycin resistance was 5.9% for both antibiotics (one isolate each). Three isolates were resistant to ceftazidime/avibactam (3/17, 17.6%) and carried *bla*_NDM-5_ (2 isolates) or *bla*_VIM-1_ (one isolate) carbapenemase gene. All the isolates were susceptible to amikacin and tigecycline.

All CS-*E. coli* isolates were susceptible to ertapenem, meropenem, ceftazidime/avibactam, piperacillin/tazobactam, amikacin, colistin, fosfomycin and tigecycline (Figure 1B). The highest resistance rates were observed for amoxicillin/clavulanic acid (36.4%, 16/44), followed by ciprofloxacin (34.1%, 15/44) and trimethoprim/sulfamethoxazole (29.5%, 13/44). Lower resistance rates were noted for gentamicin (15.9%, 7/44), ceftazidime (13.6%, 6/44), and cefepime (11.6%, 5/43).

### 3.3 Whole-genome sequencing and *in silico* analysis

#### 3.3.1 Genotyping

**Figure 2:**
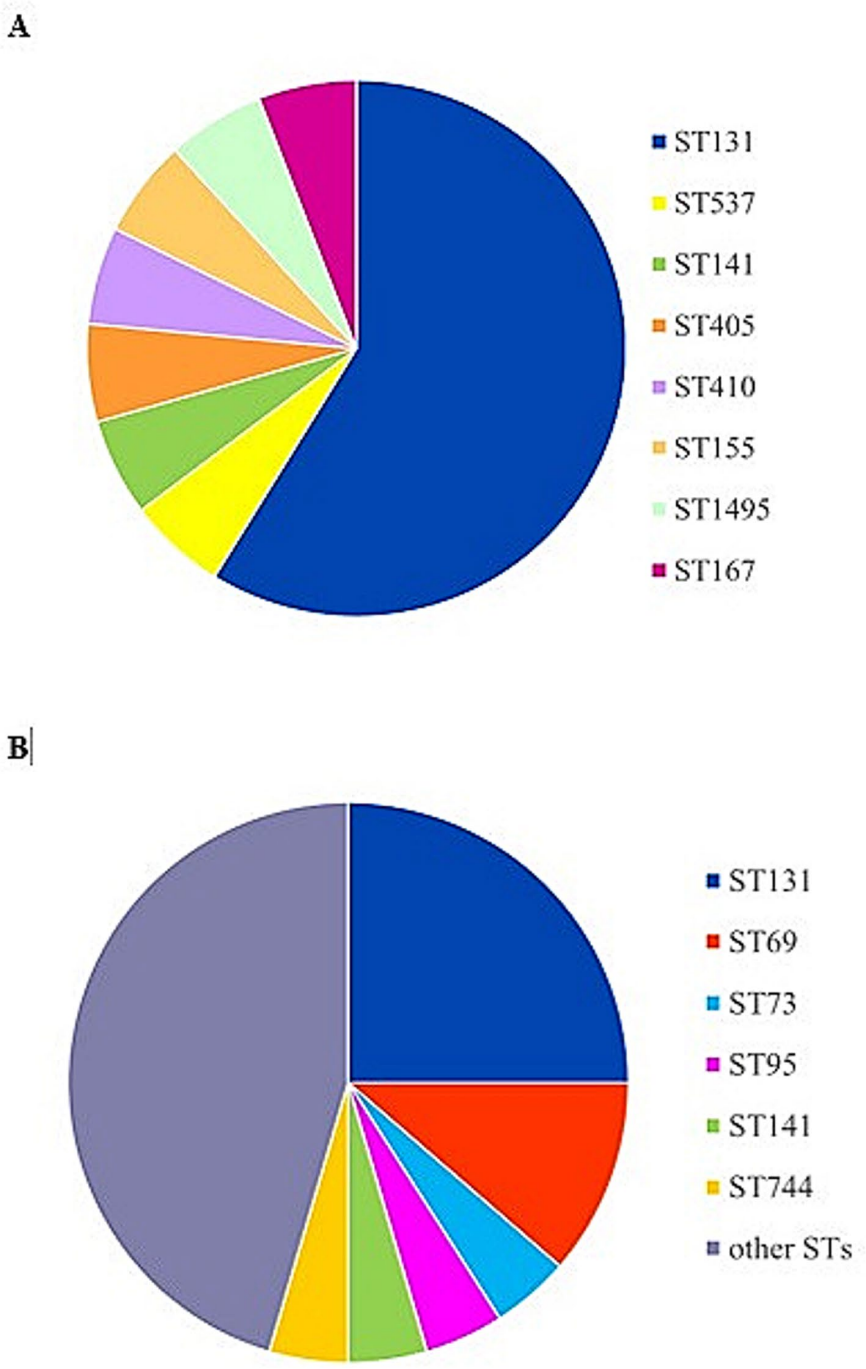
Distribution of sequence types of *E. coli* isolates from two datasets: carbapenem-resistant dataset (**A**, *N* = 17), and carbapenem-susceptible dataset (**B**, *N* = 44). STs present with less than two isolates are presented as other.

In silico analysis showed that more than half of the CR-*E. coli* isolates belonged to serotype O25: H4 (10/17, 58.8%), while CS-*E. coli* isolates belonged to 31 different serotypes, being O25: H4 (10/44, 22.7%) the most frequently detected (Supplementary Table 1). Phylogroup B2 was the most frequent in both groups (70.6% in CR- and 61.4% in CS-*E. coli* isolates, respectively), followed by phylogroups A and D (both 5.9 and 13.6%, in CR- and CS- *E. coli* isolates, respectively). Overall, we found 32 different STs among CR- and CS-*E. coli* isolates. ST131 and ST141 were the only STs detected in both groups. Seventeen CR-*E. coli* isolates belonged to 8 different STs, with one predominant ST, ST131 (*n* = 10, 58.8%) (Figure 2A). A total of 44 CS-*E. coli* isolates were grouped in 26 STs, with two more frequent STs: ST131 (*n* = 11, 25%) and ST69 (*n* = 5, 11.4%) (Figure 2B). Among CR- and CS-*E. coli* isolates, in addition to ST131 and ST69, several globally recognized high-risk lineages were detected: ST167, ST405, and ST410 appear only among CR-*E. coli* isolates, whereas ST73 and ST95 appear only among CS-*E. coli* isolates. However, each ST was represented by only one or a small number of isolates. All ST131 isolates except one belonged to CH-type 40–30, in both groups (Supplementary Table 1).

#### 3.3.2 Resistance genes

All the CR-*E. coli* isolates carried a carbapenemase gene, mainly *bla*_KPC_ (13/17, 76.4%) of which 10 isolates carried *bla*_KPC-3_ and three *bla*_KPC-2_ (Table 2). Three (17.6%) isolates harboured a metallo *β*-lactamase gene of which two were *bla*_NDM-5_ and one *bla*_VIM-1_; one (5.9%) isolate carried *bla*_OXA-181_. No isolate carried more than one carbapenemase gene.

**Table 2:** Resistome and plasmid analysis of 17 CR-*E. coli* isolates.

Isolate ID	ST	Plasmid content	Carbapenemase	ESBL	Other *β*-lactamases	Aminoglycosides	FQs	SUL	TET	Trimethoprim
IT04_R3	131	IncFIA, IncFIB, IncFII, IncI1, IncR, Col156	KPC-3	CTX-M-27	TEM-1	*aad-5*	–	*sul1; sul2*	–	*dfrA17; dfrA8*
IT23_R10	131	IncFIA, IncFIB, IncFIC, IncFII, Col156	KPC-3	–	OXA1; TEM-1	*aac(3)IIa; aac(6′)-Ib-cr*	–	–	–	*dfrA17*
IT26_R9	131	IncFIB, IncFII, Col8282	KPC-3	CTX-M-15	OXA-1	*aac(3)IIa; aac(6′)-Ib-cr*	–	–	–	–
IT37_R4	131	IncFIA, IncFIB, IncFIC, IncI1	KPC-3	CTX-M-14	OXA-1	*aac(3)IIa; aac(6′)-Ib-cr*	–	–	–	–
IT41_R4	131	IncFIB, IncFII, IncY, ColRNAI	KPC-3	-	TEM-1	*-*	–	–	*tet*(A)	–
IT45_R1	131	IncFIA, IncFIB, IncFII, Col (MG828)	KPC-3	CTX-M-15	TEM-1	*aac(3)IId*	–	*sul1; sul2*	*tet*(A)	*dfrA17*
IT45_R2	131	IncFIA, IncFIB, IncFII, Col (MG828)	KPC-3	CTX-M-15	OXA1; TEM-1	*aac(3)IId; aac(6′)-Ib-cr; ant(2″)-Ia*	–	*sul1; sul2*	*tet*(A)	*dfrA17*
IT29_R8	131	IncFIA, IncFIB, IncFIC, IncFII, Col156	KPC-2	CTX-M-15	OXA1; TEM-1	*aac(3)IIa; aac(6′)-Ib-cr*	–	*sul1*	*tet*(A)	*dfrA17*
IT32_R5	131	IncFIB, IncFII, IncX1, Col (MG828)	KPC-2	CTX-M-15	OXA1; TEM-1	*aac(6′)-Ib-cr*	*qnrB1*	–	–	*dfrA14*
IT05_R5	131	IncA/C, IncQ1, IncX3	VIM-1	SHV-12	–	*aac(6’)Ib4; aadA1; aphA15*	*qnrS1*	*sul1; sul2*	*tet*(A)	*dfrA14*
IT12_R8	141	IncFIB, IncFII, IncX1	KPC-3	–	–	–	–	–	–	–
IT46_R2	155	IncFIA, IncFIB, IncX3, IncY, Col8282	KPC-3	–	–	–	–	–	–	–
IT09_R6	167	IncFIA, IncFIB, IncFIC, IncI1, IncX4, Col (MG828)	NDM-5	CTX-M-32		*aac(3)IIa*	–	*sul1; sul2*	*tet*(A)	*dfrA1; dfrA12*
IT15_R9	405	IncFIA, IncFIB, IncFIC, IncFII, Col (BS512)	NDM-5	CTX-M-15	OXA-1	*aac(3)-IIa; aac(6′)-Ib-cr*	*qnrS1*	*sul1*	*tet*(B)	*dfrA12; dfrA17*
IT46_R3	410	IncFIA, IncFIB, IncFII, IncQ1, IncX3, IncY, Col (BS512)	OXA-181	CTX-M-15	TEM-1, CMY-2	*aac(3)IId; aac(6′)-Ibcr*	*qnrS1*	*sul1; sul2*	*tet*(B)	*dfrA17*
IT46_R1	537	IncFII, IncI1, IncX3, Col8282	KPC-3	–	–	–	–	–	–	–
IT21_R7	1,495	IncFIB, IncFIC, IncFII	KPC-2			–	–	–	–	–

ST, sequence type; FQs, fluoroquinolones; SUL, sulfamethoxazole; TET, tetracycline.

Additional plasmid-borne resistance genes, such as extended-spectrum *β*-lactamase (ESBL) genes, were found in most CR-*E. coli* isolates (11/17, 64.7%), as shown in Table 2. The most frequent ESBL gene was *bla*_CTX-M15_ (7/17, 41.2%), frequently found in association with ST131 and in combination with *bla*_KPC_ genes. Additional *β*-lactamase genes were detected in the majority of CR-*E. coli* isolates (11/17, 64.7%; Table 2).

Aminoglycoside-modifying enzymes were identified in 12 isolates (70.6%), as shown in Table 2; sulfamethoxazole/trimethoprim resistance genes *dfrA12*, *dfrA14*, and/or *dfrA17* were found in 10 isolates (10/17, 58.8%); sulphonamide resistance genes *sul1* and/or *sul2* were detected in eight isolates (8/17, 47.1%); tetracycline resistance genes *tet*(A) and *tet*(B) were detected in eight isolates (8/17, 47.1%). Plasmid-mediated resistance to fluoroquinolones was found in four isolates (three had *qnrS1* and one *qnrB1* gene). No *mcr* gene was detected. One isolate resistant to colistin had a mutation in *pmrB* gene, leading to the amino-acid substitution E123D in the PmrB protein; one isolate with resistance to fosfomycin had a mutation in the *glpT* gene with an amino-acid substitution E448K in the GlpT protein (data not shown).

Resistome analysis of CS-*E. coli* isolates was performed. Among these isolates, ESBL genes were detected in 18.2% (8/44) of the isolates, with *bla*_CTX-M-15_ accounting for the majority (7/8 isolates, 87.5% found in association with ST131), as shown in Table 3 and Supplementary Table 2. Additional *β*-lactamase genes were detected in 19/44 (43.2%) of CS-*E. coli* isolates (Table 3 and Supplementary Table 2). No *mcr* gene was detected.

**Table 3:** ESBL/other *β*-lactamase distribution in 44 carbapenem-susceptible *E. coli* isolates.

ESBL	Other *β*-lactamases	*N*	%
CTX-M-15	OXA-1	3	6.8
CTX-M-15	OXA-1-TEM-1	3	6.8
CTX-M-15	TEM-1	1	2.3
–	OXA-1	2	4.5
–	TEM-1	10	22.7
TEM-40	–	1	2.3
–	–	24	54.6

Aminoglycoside-modifying enzymes genes were found in 14 isolates (14/44, 31.8%), as shown in Supplementary Table 2. Sulfamethoxazole/trimethoprim resistance genes were found in 15 isolates (15/44, 34.1%); sulphonamide resistance genes *sul1* and/or *sul2* and/or *sul3* were detected in 16 isolates (16/44, 36.4%); tetracycline resistance genes *tet*(A), *tet*(B), and *tet*(C) were detected in 15 isolates (15/44, 34.1%). Plasmid-mediated resistance to fluoroquinolones was found in 9 isolates carrying *aac(6′)-lb-cr* gene (9/44, 20.5%) and in one isolate harbouring *qnrB6* (1/44, 2.3%; Supplementary Table 2).

#### 3.3.3 Plasmid content analysis of *E. coli* isolates

Plasmid replicon content was determined by PlasmidFinder: all CR-*E. coli* isolates harboured several plasmid replicons. Overall, IncF was the predominant replicon found in all the CR-*E. coli* isolates except one (16/17, 94.1%), followed by Col-type (13/17, 76.5%; Table 2). In particular, IncFIB was present in all except two isolates (15/17, 88.2%), IncFII in 12 isolates (12/17, 70.6%), and IncFIA in 10 isolates (10/17, 58.8%).

Besides IncF, several replicon classes (IncA/C, IncI-1, IncQ1, IncR, IncX1, IncX3, IncX4, and IncY) were found among the isolates in different combinations (Table 2). *bla*_KPC-3_ was located on a IncFII plasmid in 5/10 isolates (50%), followed by IncFIB, IncI1, IncR, IncX3 and Col (MG828) (one each). *bla*_KPC-2_ was found on IncFII (2 cases), *bla*_VIM-1_ on IncA/C, and *bla*_OXA-181_ on ColKP3. In three cases it was not possible to determine the exact location of carbapenemase gene due to the short-read sequencing method used.

Carbapenem-susceptible *E. coli* isolates harboured fewer replicons than CR-*E. coli*: IncF was found in 65.9% (29/44) of the CS-*E. coli* isolates, followed by Col-type (21/44, 47.7%; Supplementary Table 3). IncFIB was present in 24 isolates (24/44, 54.5%), IncFII in 15 isolates (15/44, 34.1%), IncFIA in 11 isolates (11/44, 25.0%). Besides IncF, other replicons classes (IncHI1A, IncB/O/K/Z, IncI-1, IncI2, IncQ1, IncN and IncX4), were found. Eight CS-*E. coli* isolates did not harbour any plasmid with the replicon scheme used.

#### 3.3.4 Virulence genes

The main virulence genes identified in *E. coli* are summarized in Table 4, according to carbapenem resistance (details in Supplementary Table 4). Several virulence genes coding for virulence factors (VFs) involved in adhesion, capsule formation, iron acquisition, and toxicity were detected. The genes encoding adhesion proteins (adhesins, fimbriae, pilin, and outer membrane proteins) were found in most strains, both in CR- and CS-*E. coli* isolates: the *fim*H gene, coding for the type 1 fimbriae, was found in 94.1 and 95.5% of isolates, respectively; *papC,* coding for outer membrane usher P fimbriae, was found in 58.8 and 54.5% of isolates, respectively. *Kps* genes, encoding the group two capsule, were detected in 70% of both groups of isolates. Several siderophores for iron acquisition were detected: *chuA*, coding for outer membrane hemin receptor, was found in 77 and 80%, respectively; *irp2*, coding for iron receptors, in 82.4 and 75%, respectively; *iucC,* coding for aerobactin synthetase, in 58.8 and 66%, respectively. Virulence genes coding for VFs related to toxicity were found in fewer isolates: *sat*, coding for a serine protease autotransporter, was found in 41 and 43%, respectively; *senB*, a plasmid-encoded enterotoxin, was found in 35.3 and 22.7%, respectively; *vat*, a vacuolating autotransporter toxin, was found only in 11.8% of CR-*E. coli* isolates.

**Table 4:** Virulence gene content, according to their functions, of 61 *E. coli* isolates (17 CR-*E. coli* and 44 CS-*E. coli*).

Virulence factor	CR-*E. coli* (*N*)	%	CS-*E. coli* (*N*)	%
Adhesins				
*fimH*	16	94.1	42	95.5
*papC*	10	58.8	24	54.5
*focC*	2	11.8	4	9.1
*sfaD*	2	11.8	0	0.0
Capsule				
*kpsE*	12	70.6	32	72.7
*neuC*	1	5.9	8	18.2
Siderophores				
*chuA*	13	76.5	35	79.5
*irp2*	14	82.4	33	75.0
*iutA*	9	52.9	29	65.9
*iroN*	3	17.6	17	38.6
*iucC*	10	58.8	29	65.9
Toxins				
*sat*	7	41.2	19	43.2
*senB*	6	35.3	10	22.7
*vat*	2	11.8	0	0.0

#### 3.3.5 Phylogenetic analysis

**Figure 3:**
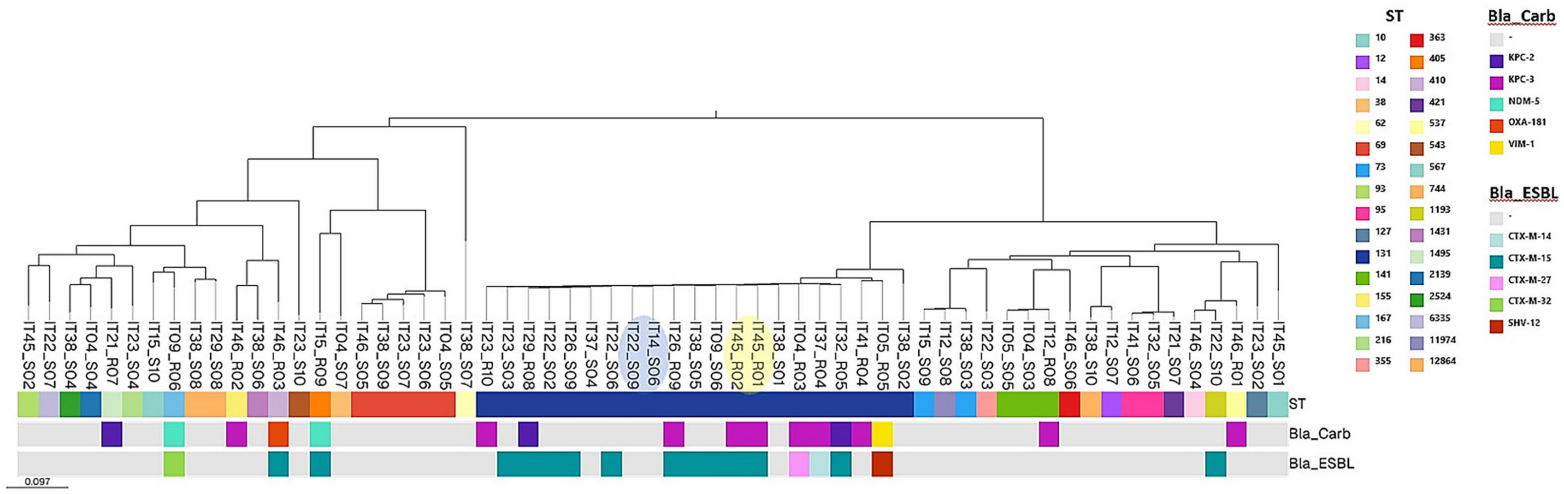
SNP-based Phylogenetic tree of 61 *E. coli* isolates. The maximum likelihood tree was rooted in a reference isolate *E. coli* strain K12 (accession no. NC_000913.2). Isolate codes (carbapenem-R or -S) are shown for each isolate. The pairs of isolates with SNP differences of 1 or 5 are highlighted in light blue and yellow, respectively.

A core genome SNP-phylogenetic tree showed high diversity amongst the 61 *E. coli* isolates (Figure 3), with a SNP distance between all isolates ranging up to 45,572 SNPs (Supplementary Table 5). ST131 clade accounted for the highest number (21/61, 34.4%) of interspersed CR- and CS-*E. coli*, including two isolates (IT23_R10 and IT23_S03, 56 SNP differences), CR- and CS-*E. coli* respectively, from the same hospital.

Carbapenem-resistant isolates clustered mainly in the ST131 clade, whereas non-ST131 CR-*E. coli* isolates were dispersed amongst susceptible isolates in the tree. The ST131 clade was characterized by the presence of carbapenemase genes in CR-*E. coli* isolates, along with multiple resistance genes and replicons (Figure 3 and Table 2). Carbapenem-susceptible comparator *E. coli* isolates belonged to different STs and had fewer resistance genes and replicons compared to CR-*E. coli* isolates (Figure 3 and Supplementary Table 2). Some isolates belonged to single-locus variants (slv) or to double-locus variants (dlv) of the ST that defines the clonal complex (CC) and were in the same branches in the tree: ST10, ST167, and ST744 belonging to clonal complex (CC) 10; ST12 and ST12864 (CC12); ST95 and ST421 (CC95); ST14, ST537, and ST1193 (CC14); ST73, ST355, and ST11974 (CC73); ST2139 and ST2524, which did not cluster in any CC (Figure 3). Two pairs of isolates belonging to ST131 were identified as closely related, showing SNP differences of 1 and 5, respectively. The first pair consisted of two isolates of CS-*E. coli* (IT22_S09 and IT14_S06, highlighted in light blue in Figure 3) that had 1 SNP difference and was found in two different cities, in the north and centre of Italy respectively, several months apart. The second pair of CR-*E. coli* isolates (IT45_R01 and IT45_R02, highlighted in yellow in Figure 3), with 5 SNP differences, was isolated one day apart in the medical unit of the same hospital, in two elderly patients with hospital acquired infections, suggesting an intra-hospital spread of this clone.

## 4 Discussion

We analysed epidemiological, microbiological and genomic data of 17 CR- and 44 CS-*E. coli* isolates collected from Italian hospitals as part of the CCRE survey in 2019. Our analysis showed that carbapenemase genes had been acquired by isolates of eight different *E. coli* STs, however, ST131 was predominant. *E. coli* ST131 is a major extraintestinal pathogenic *E. coli* (EXPEC) lineage that has driven the global spread of *bla*_CTX-M-15_ and is frequently multidrug-resistant (Mathers et al., 2015). The detection of several ST131 isolates that carry *bla*_KPC-2_ and *bla*_KPC-3_ in this study is therefore of high concern. Of note, two clusters of two ST131 isolates were identified signifying potential transmission events.

In a recent European investigation of 594 *E. coli* ST131 isolates carrying carbapenemase genes from 17 European countries (not including Italy), other carbapenemase genes such as *bla*_OXA-48_ and *bla*_OXA-244_ were much more frequent than *bla*_KPC-2_ and *bla*_KPC-3_ (Kohlenberg et al., 2024). Also, in the full CCRE survey *E. coli* dataset with 211 carbapenem-R isolates from 32 countries, only 21 isolates were found to carry *bla*_KPC_ variants (David et al., 2026), of which more than half were detected in Italy as described in this manuscript. A potential reason for the comparatively high number of *E. coli* ST131 carrying *bla*_KPC-3_ or *bla*_KPC-2_ in Italy may be local acquisition of these genes. Indeed, *bla*_KPC_ variants were the predominant carbapenemase genes in carbapenem-resistant *K. pneumoniae* circulating in the Italian healthcare system (Grundmann et al., 2017), and almost all bloodstream infections caused by carbapenemase-producing Enterobacterales were due to KPC-producing *K. pneumoniae* in the time preceding the CCRE survey (Iacchini et al., 2019).

In 2019, in the EU/EEA, the population-weighted mean carbapenem-resistance proportion in *E. coli* was reported to be 0.3% (with a country range of 0.0–0.8%, with 0.4% in Italy) (WHO Regional Office for Europe/European Centre for Disease Prevention and Control, 2022). The isolates in the CCRE survey originated mainly from elderly hospitalized patients, presenting with UTI or bloodstream infections. As reported in the literature, the elderly are at increased risk of infection by CR-*E. coli*, since they are frequently hospitalized and colonised by resistant microorganisms (Tinelli et al., 2022). CR-*E. coli* are frequently multidrug-resistant causing difficult-to-treat infections. In the present study, we found that CR-*E. coli* were resistant to several antibiotic classes: *β*-lactams, including carbapenems, sulphonamides, fluoroquinolones and aminoglycosides (only gentamycin). All isolates were susceptible to amikacin and tigecycline, in line with data from the joint WHO-ECDC/EARS-Net report published in 2022 (WHO Regional Office for Europe/European Centre for Disease Prevention and Control, 2022). Almost 20% of the isolates were resistant to ceftazidime/avibactam due to metallo *β*-lactamase production, as reported previously (Kiratisin et al., 2021). Approximately 65% of CR-*E. coli* isolates harboured ESBL genes, most frequently *bla*_CTX-M15_.

Carbapenemase genes were identified in all carbapenem-R isolates, with *bla*_KPC_ genes accounting for more than 75% of the isolates, as reported previously for Italy (Tinelli et al., 2022), and in contrast to other European countries, where the predominant carbapenemases in *E. coli* are *bla*_NDM-5_ and *bla*_OXA-48_ (WHO Regional Office for Europe/European Centre for Disease Prevention and Control, 2022). Several replicon types associated with carbapenemase genes were detected. The presence of several plasmid types that can harbour carbapenemases can favour the spread of such genes even to other species (Linkevicius et al., 2023). No strain carried the *mcr* gene, indicating that colistin resistance was not due to plasmid-mediated mechanisms.

Most CR-*E. coli* isolates belonged to ST131, as observed in previous studies, mainly associated with *bla*_KPC_ and *bla*_CTX-M-15_ (Giufrè et al., 2018a; Giufrè et al., 2018b). The presence of other international high-risk clones beside ST131 was observed, including ST167 and ST405 carrying *bla*_NDM-5_, and ST410 carrying *bla*_OXA-181_. In a previous European Survey of Carbapenemase-Producing Enterobacteriaceae (EuSCAPE) in 2013, only three CR-*E. coli* isolates were found in Italy (two belonging to ST131 and one to ST2013, a single locus variant of ST73) that did not produce any carbapenemases (Grundmann et al., 2017). In other studies, conducted at national level on infection cases, we identified CR-*E. coli* belonging to ST167 carrying *bla*_NDM-5,_ as well as CR-*E. coli* belonging to ST131carrying *bla*_VIM-1_ (Giufrè et al., 2018a; Giufrè et al., 2018b).

The CCRE survey demonstrated that ST131 is one of the predominant international high-risk *E. coli* clones associated with carbapenemase production across Europe (David et al., 2026). The widespread distribution of ST131 observed in our study is consistent with the broader Mediterranean and European epidemiology, highlighting its successful dissemination and major role in the global spread of antimicrobial resistance. In Western Mediterranean countries, including Spain, France, as well as Portugal, OXA-48-like carbapenemases predominate, while NDM-producing isolates have become increasingly frequent, reflecting the evolving epidemiology of carbapenem resistance. These findings support the role of cross-border spread, international travel, and healthcare-associated transmission in the dissemination of carbapenem-resistant *E. coli* (David et al., 2026). Similarly, in North Africa, OXA-48 is endemic in Algeria and Tunisia, whereas NDM has emerged as an important resistance determinant in Morocco and Egypt (Macesic et al., 2026). Notably, the pandemic ST131 clone has been reported in several North African countries, including Algeria, Tunisia, Morocco, and Egypt, supporting the circulation of this high-risk lineage across the Mediterranean region (Abi et al., 2026).

The prevalence of ST167, ST405, ST410, ST361, and ST648 in 874 *E. coli* isolates carrying *bla*_NDM-5_ was reported from 13 European Union or European Economic Area countries over 2012–2022 (Linkevicius et al., 2023). At present, the occurrence and spread of CR-*E. coli* has been documented across many different regions in the world (Huang et al., 2024). This indicates a concerning increase in the circulation of high-risk *E. coli* clones producing carbapenemases, including OXA-181, the most common OXA-48-like variant. Although in our study only one OXA-181 *E. coli* isolate was found, the dissemination of this carbapenemase is worrisome, as it has been reported globally in both healthcare and community settings, as well as in animals and the environment (Mairi et al., 2018). A recent Italian study also confirmed the spread of OXA-181-producing *E. coli* among pig and veal calf holdings (Carfora et al., 2022), emphasizing the importance of genomic surveillance from a One Health perspective.

Carbapenem-susceptible *E. coli* isolates were more heterogeneous and belonged to several different STs, even though ST131 and ST69 accounted for a third of the isolates.

In our previous study on ESBLs, we documented the predominance of the pandemic clone ST131, harbouring ESBLs of the CTX-M-15 type and showing additional resistance to several antibiotics. Worldwide, numerous sequence types are described and the most frequently reported STs are ST131, ST69, ST10, ST405, ST38, ST95, ST648, ST73 and ST1193, which have been detected in both hospital- and community-associated infections (Manges et al., 2019; Shaik et al., 2017).

Considering all the European *E. coli* isolates collected for the CCRE survey, a phylogeny study showed a high diversity among all the *E. coli* isolates (David et al., 2026). Only 23 of the 493 *E. coli* isolates (4.7%) had less than or equal to 17 SNPs with other similar isolates, with only five geographically linked pairs. CR-*E. coli* isolates from the studied European countries largely occurred as singletons or in small clusters in the global phylogenies of CR-*E. coli*, demonstrating numerous imports into the European countries with minimal onward local transmission (David et al., 2026).

A limitation of this study is that the inclusion process of the CCRE survey consisted of collecting the first 10 non-duplicated consecutive isolates of CR- *K. pneumoniae* or *E. coli* combined. As *K. pneumoniae* considerably outnumbered *E. coli*, only few CR-*E. coli* isolates were obtained from the participating hospitals; if *E. coli* had been collected independently of *K. pneumoniae*, a higher number of CR-*E. coli* isolates could have been obtained. Nevertheless, the isolates collected during the study period provided a snapshot of the CR-*E. coli* clones circulating in Italy in 2019. Further studies are ongoing to evaluate any change that occurred in recent years.

In conclusion, in Italy, in the study period, carbapenem resistance in *E. coli* remained infrequent, comparatively to resistance in *K. pneumoniae*, in line with the low prevalence reported at the EU/EEA level (European Centre for Disease Prevention and Control and World Health Organization, 2023). However, our findings highlight a concerning epidemiological scenario characterized by the emergence of carbapenemase-producing isolates within well-established high-risk lineages, particularly the predominant ST131 clone. The identification of ST131 isolates carrying *bla*_KPC_ variants, together with the detection of additional international high-risk clones (including ST167, ST405, and ST410) harboring *bla*_NDM-5_ or *bla*_OXA-181_, demonstrates that carbapenemase genes have been established across multiple successful *E. coli* lineages in Italy. Continuous genomic surveillance strengthened infection prevention and control measures, prudent antimicrobial stewardship, and integrated monitoring across human and veterinary sectors are essential to contain the spread of carbapenem-resistant *E. coli* in line with a One Health approach.

## Data Availability

The datasets presented in this study can be found in online repositories. The names of the repository/repositories and accession number(s) can be found in the article/Supplementary material.

## Footnotes

1 https://www.genomicepidemiology.org/services/ accessed on 8 April 2025.

2 https://www.ncbi.nlm.nih.gov/pathogens/antimicrobial-resistance/AMRFinder/ accessed on 8 April 2025.

3 https://pathogen.watch/en accessed on 22/06/2026.

4 https://usegalaxy.eu/ accessed on 22/06/2026.

5
https://cge.food.dtu.dk/services/CSIPhylogeny/

6 https://microreact.org/ accessed on 15 April 2025.

## Members of the Italian CCRE working group

Alessandria, ROCCHETTI ANDREA (Azienda Ospedaliera Nazionale SS. Antonio e Biagio e Cesare Arrigo); Ancona, ORECCHIONI FRANCESCA (Ospedale Torrette Umberto I); Asti, CONCIALDI ERIKA, VADA IVANA (Ospedale Cardinal Massaia); Bari, MOSCA ADRIANA (Università degli Studi di Bari); Bologna, AMBRETTI SIMONE (Policlinico S. Orsola Malpighi); Bolzano, ASCHBACHER RICHARD, PAGANI ELISABETTA (Azienda Sanitaria dell’Alto Adige-Comprensorio Sanitario di Bolzano); Caserta, GRECO RITA (Azienda Ospedaliera S. Anna e S. Sebastiano); Catania, IMBRIANI ALIDA, STEFANI STEFANIA (Azienda Ospedaliero-Universitaria Policlinico Vittorio Emanuele); Cesena (FC), PEDNA MARIA FEDERICA (Laboratorio Unico-Centro Servizi AUSL della Romagna); Cittadella (PD), SARTORE PAOLA, BIASIO GIANFRANCO, POGGIANA LUCIA (Ospedale Civile di Cittadella-ULSS6-Euganea); Cona (FE), BAROZZI AGOSTINO, CARILLO CARMELINA (Azienda Ospedaliero-Universitaria di Ferrara); Cosenza, GIRALDI CRISTINA, DODARO SAVERIA, GRECO FRANCESCA, MAURO MARIA VITTORIA (Azienda Ospedaliera Cosenza-Presidio Ospedaliero Annunziata); Firenze, RICCOBONO ELEONORA, PECILE PATRIZIA, BACCANI ILARIA, ROSSOLINI GIAN MARIA (Azienda Ospedaliero-Universitaria Careggi); Lecco, MAURI CAROLA (Ospedale A. Manzoni - ASST di Lecco); Mestre (VE), SCARPARO CLAUDIO (Ospedale dell’Angelo-ULSS 3 Serenissima); Milano, CICHERO PAOLA, KOROVESHI BRISEJDA (Ospedale S. Raffaele Milano), GRANCINI ANNA (Fondazione IRCCS Ca′ Granda Ospedale Maggiore Policlinico); Napoli, AMATO GERARDINO (Azienda Ospedaliera A. Cardarelli), BERNARDO MARIANO (Azienda Ospedaliera Specialistica dei Colli), CATANIA MARIA ROSARIA (Azienda Ospedaliera Universitaria Federico II); Palermo, GIAMMANCO ANNA, FASCIANA TERESA (Università degli Studi di Palermo); Perugia, MENCACCI ANTONELLA (Ospedale Santa Maria della Misericordia); Roma, DI CARO ANTONINO (Ospedale Lazzaro Spallanzani), MELEDANDRI MARCELLO (ASL Roma 1 Presidio S. Filippo Neri), PARISI GABRIELLA (Ospedale San Camillo); Rovereto (TN), GUALDI PAOLA (Ospedale *S. Maria* del Carmine); Salerno, REGA MARIA ROSARIA (Azienda Ospedaliera S. Giovanni di Dio e Ruggi D’Aragona); Sanremo, DUSI PIER ANDREA (Ospedale di Sanremo ASL 1 IMPERIESE); Siena, CUSI MARIA GRAZIA (Azienda Ospedaliera Universitaria Senese-Policlinico Santa Maria Alle Scotte); Torino, GHIBAUDO DAVIDE (Azienda Ospedaliero-Universitaria Città della Salute e della Scienza-Ospedale Molinette); Treviso, CAMPION LAURA (Ospedale *S. Maria* di Ca′Foncello); Vercelli, MILANO FULVIA (Ospedale Sant’Andrea).
