# Supplementary material for: Whole-genome sequencing and molecular analysis of carbapenem-resistant Escherichia coli from clinical samples collected in the framework of the EURGen-Net CCRE survey, Italy 2019
Source: Front Microbiol. 2026 Jul 24;17:1875003. doi: 10.3389/fmicb.2026.1875003 (PMC13447248; doi:10.3389/fmicb.2026.1875003)
Supplement: Supplementary file 1 [file Table_1.docx]

Supplementary Material

Supplementary table 1. Genotyping of 61 Italian *E. coli* isolates (17 CR-*E. coli* and 44 CS-*E. coli*)

| **Isolate ID** | **Serotype** | **Phylogroup** | **CH-type** | **ST** | **cgMLST** |
| --- | --- | --- | --- | --- | --- |
| **CR-E. coli** |  |  |  |  |  |
| IT04_R03 | O25:H4 | B2 | 40-30 | 131 | 188566 |
| IT05_R05 | O25:H4 | B2 | 40-298 | 131 | 188569 |
| IT09_R06 | O8:H9 | A | 11-0 | 167 | 188536 |
| IT12_R08 | O2:H6 | B2 | 52-117 | 141 | 188546 |
| IT15_R09 | O102:H6 | D | 37-27 | 405 | 188520 |
| IT21_R07 | -:H4 | nd* | 11-94 | 1495 | 188574 |
| IT23_R10 | O25:H4 | B2 | 40-30 | 131 | 188486 |
| IT26_R09 | O25:H4 | B2 | 40-30 | 131 | 188525 |
| IT29_R08 | O25:H4 | B2 | 40-30 | 131 | 29037 |
| IT32_R05 | O25:H4 | B2 | 40-30 | 131 | 188499 |
| IT37_R04 | O25:H4 | B2 | 40-30 | 131 | 188565 |
| IT41_R04 | O25:H4 | B2 | 40-30 | 131 | 188480 |
| IT45_R01 | O25:H4 | B2 | 40-30 | 131 | 188570 |
| IT45_R02 | O25:H4 | B2 | 40-30 | 131 | 188570 |
| IT46_R01 | O75:H5 | B2 | 14-144 | 537 | 188567 |
| IT46_R02 | -:H10 | B1 | 4-121 | 155 | 188551 |
| IT46_R03 | O8:H9 | C | 4-24 | 410 | 188553 |
| **CS-E. coli** |  |  |  |  |  |
| IT04_S03 | O2:H6 | B2 | 52-5 | 141 | 188564 |
| IT04_S04 | O156:H30 | nd | 7-86 | 2139 | 188556 |
| IT04_S05 | O15:H6 | D | 35-27 | 69 | 188543 |
| IT04_S07 | O1:H15 | D | 26-65 | 38 | 188554 |
| IT05_S05 | O2:H6 | B2 | 52-5 | 141 | 188542 |
| IT09_S06 | O25:H4 | B2 | 40-30 | 131 | 188527 |
| IT12_S07 | O4:H1 | B2 | 13-106 | 12 | 188498 |
| IT12_S08 | O6:H1 | B2 | 24-10 | 11974 | 188547 |
| IT14_S06 | O25:H4 | B2 | 40-30 | 131 | 188496 |
| IT15_S09 | O18:H1 | B2 | 24-10 | 73 | 188066 |
| IT15_S10 | O8:H17 | A | 11-93 | 10 | 188522 |
| IT21_S07 | O1:H7 | B2 | 38-92 | 421 | 188592 |
| IT22_S02 | O25:H4 | B2 | 40-30 | 131 | 33630 |
| IT22_S03 | O120:H5 | B2 | 24-1473 | 355 | 188624 |
| IT22_S06 | O25:H4 | B2 | 40-30 | 131 | 188628 |
| IT22_S07 | -:H2 | A | 27-30 | 6335 | 188590 |
| IT22_S09 | O25:H4 | B2 | 40-30 | 131 | 188496 |
| IT22_S10 | O75:H5 | B2 | 14-64 | 1193 | 199125 |
| IT23_S02 | O6:H31 | B2 | 14-2 | 127 | 188568 |
| IT23_S03 | O25:H4 | B2 | 40-30 | 131 | 188561 |
| IT23_S04 | O7:H4 | A | 11-69 | 216 | 188560 |
| IT23_S06 | O15:H18 | D | 35-27 | 69 | 188563 |
| IT23_S07 | O15:H1 | D | 35-27 | 69 | 188557 |
| IT23_S10 | O169:H9 | E | 136-331 | 543 | 188548 |
| IT26_S09 | O25:H4 | B2 | 40-30 | 131 | 29037 |
| IT29_S08 | -:H9 | A | 11-54 | 744 | 188586 |
| IT32_S05 | O1:H7 | B2 | 38-30 | 95 | 188071 |
| IT37_S04 | O25:H4 | B2 | 40-30 | 131 | 188526 |
| IT38_S01 | O25:H4 | B2 | 40-30 | 131 | 188629 |
| IT38_S02 | O16:H5 | B2 | 40-42 | 131 | 188661 |
| IT38_S03 | O2/O50:H1 | B2 | 24-10 | 73 | 188541 |
| IT38_S04 | O6:H33 | A | 7-53 | 2524 | 188532 |
| IT38_S05 | O25:H4 | B2 | 40-30 | 131 | 188533 |
| IT38_S06 | O8:H30 | B1 | 65-32 | 1431 | 188599 |
| IT38_S07 | -:H45 | F | 33-44 | 62 | 188608 |
| IT38_S08 | -:H10 | A | 11-54 | 744 | 188600 |
| IT38_S09 | O107/O117:H4 | D | 35-27 | 69 | 188601 |
| IT38_S10 | O4:H5 | B2 | 13-2907 | 12864 | 188603 |
| IT41_S06 | O1:H7 | B2 | 38-1193 | 95 | 188545 |
| IT45_S01 | O83:H33 | B2 | 43-13 | 567 | 188549 |
| IT45_S02 | O5:H10 | nd | 11-0 | 93 | 188580 |
| IT46_S04 | O18:H5 | B2 | 14-27 | 14 | 188577 |
| IT46_S05 | O107/O117:H4 | D | 35-27 | 69 | 69345 |
| IT46_S06 | O46:H31 | B2 | 38-5 | 363 | 188535 |

*nd-not determined

**Supplementary Table 2.** Resistome analysis of 44 carbapenem-susceptible *E. coli* isolates.

| Isolate ID | ST | ESBL | Other β-lactamases | Aminoglycosides | Fluoroquinolones | Sulfamethoxazole | Tetracycline | Trimethoprim |
| --- | --- | --- | --- | --- | --- | --- | --- | --- |
| IT15_S10 | 10 | - | TEM-1 |  | *-* | *sul2* | *-* | *dfrA1* |
| IT12_S07 | 12 | TEM-40 |  | *-* | *-* | *-* | *-* | *-* |
| IT46_S04 | 14 | - | TEM-1 | *-* | *-* | *-* | *-* | *-* |
| IT04_S07 | 38 | - |  | *-* | *-* | *-* | *-* | *-* |
| IT38_S07 | 62 | - |  | *-* | *-* | *-* | *-* | *-* |
| IT04_S05 | 69 | - |  | *-* | *-* | *sul2* | *tet*(B) | *-* |
| IT23_S06 | 69 | - | TEM-1 | *aac(6')-Ib-cr;aadA; aadA16;aph3-Ia* | *qnrB6* | *sul1; sul2* | *tet*(A) | *dfrA1* |
| IT46_S05 | 69 | - |  | *-* | *-* | *-* | *-* | *-* |
| IT23_S07 | 69 | - |  |  | *-* | *-* | *-* | *-* |
| IT38_S09 | 69 | - |  |  | *-* | *-* | *-* | *-* |
| IT15_S09 | 73 | - | - | *-* | *-* | *-* | *-* | *-* |
| IT38_S03 | 73 | - | - | *-* | *-* | *-* | *-* | *-* |
| IT45_S02 | 93 | - | - | *-* | *-* | *-* | *-* | *-* |
| IT41_S06 | 95 | - | TEM-1 | *-* | *-* | *-* | *-* | *dfrA5* |
| IT32_S05 | 95 | - | TEM-1 |  | *-* | *sul2* | *-* | *dfrA5* |
| IT23_S02 | 127 | - | - |  | *-* | *-* | *-* | *-* |
| IT26_S09 | 131 | CTX-M-15 | OXA-1 | *aac(3)-IIa;*  *aac(6')-Ib-cr;aadA5* | *-* | *sul1* | *tet*(A) | *dfrA17* |
| IT23_S03 | 131 | CTX-M-15 | OXA-1 | *aac(6')-Ib-cr; aadA5* | *-* | *sul1* | *tet*(A) | *dfrA17* |
| IT37_S04 | 131 | - | - | *aac(3)-IIa;aadA5* | *-* | *sul1* | *-* | *dfrA17* |
| IT09_S06 | 131 | CTX-M-15 | OXA-1; TEM-1 | *aac(3)-IId;*  *aac(6')-Ib-cr; aadA5;* | *-* | *sul1;sul2* | *tet*(A) | *dfrA17* |
| IT14_S06 | 131 | - | OXA-1 | *aac(6')-Ib-cr5* | *-* | *-* | *tet*(A) | *-* |
| IT22_S02 | 131 | CTX-M-15 | OXA-1; TEM-1 | *aac(6')-Ib-cr5;*  *aac(3)-IIe* | *-* | *sul2* | *tet*(A) | *-* |
| IT22_S06 | 131 | CTX-M-15 | OXA-1; TEM-1 | *aac(6')-Ib-cr5;*  *aac(3)-IIe* | *-* | *sul2* | *-* | *-* |
| IT22_S09 | 131 | - | OXA-1 | *aac(6')-Ib-cr5* | *-* | *-* | *tet*(A) | *-* |
| IT38_S01 | 131 | - | TEM-1 | *-* | *-* | *sul1;sul2* | *tet*(A) | *dfrA17* |
| IT38_S02 | 131 | - | TEM-1 | *aac(3)-IId* | *-* | *-* | *-* | *-* |
| IT38_S05 | 131 | CTX-M-15 | TEM-1 | *aph3-Ia* | *-* | *sul1* | *tet*(A) | *dfrA12* |
| IT05_S05 | 141 | - | - | *-* | *-* | *-* | *-* | *-* |
| IT04_S03 | 141 | - | - | *-* | *-* | *-* | *-* | *-* |
| IT23_S04 | 216 | - |  | *-* | *-* | *-* | *-* | *-* |
| IT22_S03 | 355 | - | - | *-* | *-* | *-* | *-* | *-* |
| IT46_S06 | 363 | - | - | *-* | *-* | *-* | *-* | *-* |
| IT21_S07 | 421 | - | - | *-* | *-* | *-* | *-* | *-* |
| IT23_S10 | 543 | - |  | *-* | *-* | *-* | *tet*(A) | *dfrA1* |
| IT45_S01 | 567 | - | - | *-* | *-* | *-* | *tet*(C) | *-* |
| IT29_S08 | 744 | - | TEM-1 | *aadA2;aadA5;*  *aadA;aph3-Ia* | *-* | *sul1;sul2;sul3* | *tet*(B) | *dfrA12;dfrA17* |
| IT38_S08 | 744 | - | TEM-1 | *aadA5;aph(3')-Ia* | *-* | *sul2* | *tet*(B) | *dfrA17* |
| IT22_S10 | 1193 | CTX-M-15 | OXA-1 | *aac(6')-Ib-cr5* | *-* | *sul2* | *tet*(B) | *dfrA17* |
| IT38_S06 | 1431 | - |  | *-* | *-* | *-* | *-* | *-* |
| IT04_S04 | 2139 | - |  | *-* | *-* | *-* | *-* | *-* |
| IT38_S04 | 2524 | - |  | *-* | *-* | *-* | *-* | *-* |
| IT22_S07 | 6335 | - |  | *-* | *-* | *-* | *-* | *-* |
| IT12_S08 | 11974 | - | TEM-1 |  | *-* | *sul2* | *-* | *dfrA14* |
| IT38_S10 | 12864 | - | - | *-* | *-* | *-* | *-* | *-* |

**Supplementary Table 3.** Plasmid content of 44 carbapenem-susceptible *E. coli*

| Isolate ID | ST | IncA/C | IncFIA | IncFIB | IncFIC | IncFII | IncHI1A/B | IncI1 | IncI2 | IncN | IncQ1 | IncB/O/K/Z | IncR | IncX | IncY | Col |
| --- | --- | --- | --- | --- | --- | --- | --- | --- | --- | --- | --- | --- | --- | --- | --- | --- |
| IT26_S09 | 131 |  | x | x | x |  |  |  |  |  |  |  |  |  |  |  |
| IT23_S03 | 131 |  | x | x | x |  |  |  |  |  |  |  |  |  |  | x |
| IT37_S04 | 131 |  | x | x | x |  |  |  |  |  |  |  |  |  |  | x |
| IT09_S06 | 131 |  |  | x |  | x |  |  |  |  |  |  |  | x |  | x |
| IT45_S01 | 567 |  |  | x |  | x |  |  |  |  |  |  |  |  |  | x |
| IT23_S02 | 127 |  |  | x |  | x |  |  |  |  |  |  |  |  |  | x |
| IT46_S04 | 14 |  |  | x | x |  |  |  |  |  |  |  |  |  |  | x |
| IT12_S07 | 12 |  |  |  |  |  |  |  |  |  |  | x |  |  |  |  |
| IT21_S07 | 421 |  |  | x | x |  |  |  |  |  |  |  |  |  |  |  |
| IT41_S06 | 95 |  |  | x |  |  |  |  |  |  |  |  |  |  |  | x |
| IT32_S05 | 95 |  |  | x |  | x |  |  |  |  | x |  |  |  |  | x |
| IT15_S09 | 73 |  |  |  |  |  |  |  |  |  |  |  |  |  |  |  |
| IT12_S08 | 11974 |  |  |  |  |  |  |  |  |  |  |  |  |  |  | x |
| IT46_S06 | 363 |  |  |  |  |  |  |  |  |  |  |  |  |  |  |  |
| IT05_S05 | 141 |  |  |  |  |  |  |  |  |  |  |  |  |  |  |  |
| IT04_S03 | 141 |  |  |  |  |  |  |  |  |  |  |  |  |  |  |  |
| IT04_S07 | 38 |  |  |  |  |  |  |  |  |  |  |  |  |  |  | x |
| IT04_S05 | 69 |  | x | x | x |  |  |  |  |  |  |  |  |  |  | x |
| IT23_S06 | 69 |  |  | x | x |  |  |  |  | x | x | x |  |  |  |  |
| IT46_S05 | 69 |  |  |  |  |  |  |  |  |  |  |  |  |  |  | x |
| IT23_S07 | 69 |  | x | x | x | x |  |  |  |  |  |  |  |  |  | x |
| IT45_S02 | 93 |  |  | x | x |  |  | x |  |  |  |  |  |  |  | x |
| IT23_S04 | 216 |  |  |  |  |  | x |  |  |  |  |  |  |  |  | x |
| IT04_S04 | 2139 |  |  |  |  |  |  |  |  |  |  |  |  |  |  |  |
| IT29_S08 | 744 |  |  | x | x |  |  |  |  |  | x |  |  |  |  |  |
| IT15_S10 | 10 |  |  | x |  | x |  | x |  |  |  | x |  |  |  |  |
| IT14_S06 | 131 |  | x | x |  | x |  |  |  |  |  |  |  |  |  |  |
| IT22_S02 | 131 |  | x | x |  | x |  |  |  |  |  |  |  |  |  | x |
| IT22_S03 | 355 |  |  | x |  | x |  |  |  |  |  |  |  |  |  | x |
| IT22_S06 | 131 |  | x | x |  |  |  |  |  |  |  | x |  |  |  |  |
| IT22_S07 | 6335 |  |  |  |  |  |  |  |  |  |  |  |  |  |  |  |
| IT22_S09 | 131 |  | x | x |  |  |  |  |  |  |  |  |  |  |  |  |
| IT22_S10 | 1193 |  | x |  |  |  |  |  |  |  | x |  |  |  |  | x |
| IT23_S10 | 543 |  |  |  |  |  |  |  |  |  |  |  |  |  |  |  |
| IT38_S01 | 131 |  |  |  |  | x |  |  | x |  |  |  |  | x |  |  |
| IT38_S02 | 131 |  |  |  |  | x |  |  |  |  |  |  |  |  |  | x |
| IT38_S03 | 73 |  |  |  |  |  |  |  |  |  |  |  |  |  |  | x |
| IT38_S04 | 2524 |  |  | x |  |  |  |  |  |  |  |  |  |  |  |  |
| IT38_S05 | 131 |  | x |  |  | x |  |  |  |  |  |  |  |  |  |  |
| IT38_S06 | 1431 |  |  |  |  |  |  |  |  |  |  |  |  | x |  |  |
| IT38_S07 | 62 |  |  |  |  | x |  |  |  |  |  |  |  | x |  | x |
| IT38_S08 | 744 |  |  | x |  | x |  | x | x |  | x |  |  |  |  |  |
| IT38_S09 | 69 |  |  |  |  |  |  |  |  |  |  |  |  |  |  |  |
| IT38_S10 | 12864 |  |  | x |  | x |  |  |  |  |  |  |  |  |  |  |

**Supplementary Table 6.** Sample names and run accession numbers of 61 Italian *E. coli* isolates (44 CS-*E. coli* and 17 CR-*E. coli*) under study, included in the project no. PRJEB39943.

| **Sample name** | **Run accession** |
| --- | --- |
| IT04_R03 | ERR8773443 |
| IT04_S03 | ERR8773456 |
| IT04_S04 | ERR8773438 |
| IT04_S05 | ERR8774098 |
| IT04_S07 | ERR8774014 |
| IT05_R05 | ERR8773452 |
| IT05_S05 | ERR8773641 |
| IT09_R06 | ERR8773365 |
| IT09_S06 | ERR8773565 |
| IT12_R08 | ERR8773915 |
| IT12_S07 | ERR8773310 |
| IT12_S08 | ERR8773853 |
| IT14_S06 | ERR8773214 |
| IT15_R09 | ERR8773341 |
| IT15_S09 | ERR8773644 |
| IT15_S10 | ERR8773639 |
| IT21_R07 | ERR8773483 |
| IT21_S07 | ERR8774547 |
| IT22_S02 | ERR8774467 |
| IT22_S03 | ERR8774462 |
| IT22_S06 | ERR8774410 |
| IT22_S07 | ERR8774542 |
| IT22_S09 | ERR8774532 |
| IT22_S10 | ERR8773489 |
| IT23_R10 | ERR8773177 |
| IT23_S02 | ERR8773468 |
| IT23_S03 | ERR8774176 |
| IT23_S04 | ERR8774171 |
| IT23_S06 | ERR8774162 |
| IT23_S07 | ERR8774119 |
| IT23_S10 | ERR8773434 |
| IT26_R09 | ERR8773610 |
| IT26_S09 | ERR8773426 |
| IT29_R08 | ERR8773233 |
| IT29_S08 | ERR8774603 |
| IT32_R05 | ERR8773270 |
| IT32_S05 | ERR8773938 |
| IT37_R04 | ERR8773409 |
| IT37_S04 | ERR8773559 |
| IT38_S01 | ERR8774264 |
| IT38_S02 | ERR8774259 |
| IT38_S03 | ERR8773808 |
| IT38_S04 | ERR8773803 |
| IT38_S05 | ERR8773799 |
| IT38_S06 | ERR8774667 |
| IT38_S07 | ERR8774665 |
| IT38_S08 | ERR8774663 |
| IT38_S09 | ERR8774645 |
| IT38_S10 | ERR8774643 |
| IT41_R04 | ERR8773126 |
| IT41_S06 | ERR8774021 |
| IT45_R01 | ERR8773477 |
| IT45_R02 | ERR8773475 |
| IT45_S01 | ERR8773960 |
| IT45_S02 | ERR8774191 |
| IT46_R01 | ERR8773466 |
| IT46_R02 | ERR8774093 |
| IT46_R03 | ERR8774087 |
| IT46_S04 | ERR8774041 |
| IT46_S05 | ERR8773868 |
| IT46_S06 | ERR8773863 |
